# The bibliometric analysis of documents concerning the relationship between the microbiota and urological malignancies

**DOI:** 10.1099/jmm.0.002041

**Published:** 2025-07-02

**Authors:** Uygar Bağcı, Özlem Ulusan Bağcı

**Affiliations:** 1 Department of Pediatric Urology, Ankara Etlik City Hospital, Ankara, Turkey; 2 Division of Parasitology, Department of Microbiology, Faculty of Medicine, Ankara University, Ankara, Turkey

**Keywords:** bibliometric, bladder, cancer, microbiota, prostate, renal

## Abstract

**Introduction.** The microbiota, which has a major impact on both health and illness, has recently become one of the most popular research topics.

**Hypothesis/Gap statement.** To the best of our knowledge, no research has undertaken a bibliometric analysis of publications examining the connection between microbiome and urological cancer to date. In this respect, it is thought that our study will contribute to the literature.

**Aim.** The purpose of this study is to raise awareness of the topic by performing a bibliometric analysis of the publications examining the connection between the microbiota and the most common urological cancers, including bladder, prostate, and kidney cancers.

**Methodology.** All publications about prostate, renal and bladder cancers and microbiota indexed in Web of Science between 2000 and 2024 were included in the study.

**Results.** A total of 310 publications were obtained. Before 2018, there were only three or fewer publications annually; however, following 2018, the number of publications increased rapidly, reaching a peak of 77 in 2024. The USA led with 98 (31.61%) documents, followed by China (60, 19.35%) and Italy (31, 10%). With 19 publications, Hirotsugu Uemura is the most contributing author, followed by Norio Nonomura with 17. Prostate cancer accounted for 45.48% of the publications, bladder cancer for 36.77% and kidney malignancies for 17.64%.

**Conclusion.** Despite the fact that microbiota has been known for 80 years, research on the connection between microbiota and cancer accelerated after the completion of the Human Microbiome Project. The number of studies examining the connection between urological cancer and microbiota peaked in 2024 and is probably going to rise. More research is required on this topic, since the correlation between microbiota and especially prostate and bladder malignancies raises the possibility that variations in microbiota may be utilized in diagnosis, treatment and prognosis.

## Introduction

The term microbiota refers to all of the bacteria, viruses, fungi, parasites and archaea that inhabit the body. With 150 times as many genes and 10 times as many cells as the body, the microbiota is regarded as a hidden organ [1]. The intestines, which are home to the great majority of micro-organisms, are the first place that comes to mind when considering microbiota. The skin, mouth cavity, respiratory tract, urogenital area and all mucosal surfaces are less likely to have microbes [2,3]. Although microbiota and microbiome are often used interchangeably, they are actually different terms. While microbiota refers to the community of micro-organisms, microbiome is the totality of micro-organisms and their genes [2]. The micro-organisms that comprise the microbiota differ from one individual to another and may also change within the same individual depending on environmental factors, diet and age. In recent years, there has been an increase in the number of studies investigating the relationship between microbiota and diseases. Microbiota can cause carcinogenesis through processes such as the production of carcinogenic compounds, genotoxic effects, inactivation of tumour suppressor genes, induction of local inflammatory responses and immune system suppression. Moreover, microbiota components affect the treatment process by altering the efficacy of chemotherapeutics and immunotherapeutics [4].

When the causes of death in the world are examined, it is noteworthy that cancers come second after cardiovascular diseases [5]. Kidney, bladder and prostate cancers are the most common urological malignancies. Bladder cancer is more prominent in the male gender, and while it ranks fourth among the most frequently diagnosed cancers in the world in the male gender, it ranks eighth among the cancers that cause the most deaths. Nevertheless, it is not one of the top ten causes of cancer and mortality for women, according to GLOBOCAN (The Global Cancer Observatory: Cancer Today) data published in 2024 [5]. Renal cell carcinoma (RCC) is the sixth most commonly diagnosed cancer in men and the ninth most common cancer in women worldwide. According to 2024 data, RCC is not among the 10 most common causes of cancer death in both sexes, and according to 2022 data, it ranks 16th among cancer-related causes of death [5,6]. Prostate cancer is the most commonly diagnosed cancer in men worldwide and the second most common cause of death [5].

Bibliometric analysis examines the number of publications, the number of citations, the collaboration of authors and the distribution of written documents by subject in order to determine changes and developments in the scientific literature and to identify trends in publications [7]. In the presented study, we aimed to raise awareness about the microbiota in the aetiology of cancer and to create an infrastructure for prospective research on this subject by bibliometric analysis of the relationship between microbiota and the most common and most popular urological cancers.

## Methods

All publications indexed in the Web of Science (WOS) database between 2000 and 2024 that contained the keywords ‘bladder tumor/tumour/cancer/carcinoma/neoplasm microbiota/microbiome’, ‘urothelial carcinoma microbiota/microbiome’, ‘transitional cell carcinoma/tumor/tumour microbiota/microbiome’, ‘prostate cancer microbiota/microbiome’, or ‘bladder/renal cancer/tumour urobiome’ were included in the study. Duplicate publications were manually excluded. The publications were downloaded to the computer in plain text file format and then transferred to the VOSviewer 1.6.20 program. The WOS and VOSviewer programs were used to examine the most cited keywords in the documents, the most published countries/authors/journals and the most cited countries/journals/authors/articles. The Microsoft Office Excel 17 application was used to perform data calculations and create tables. Ethics committee approval was not obtained because the study did not use any human or animal material.

## Results

**Fig. 1.**
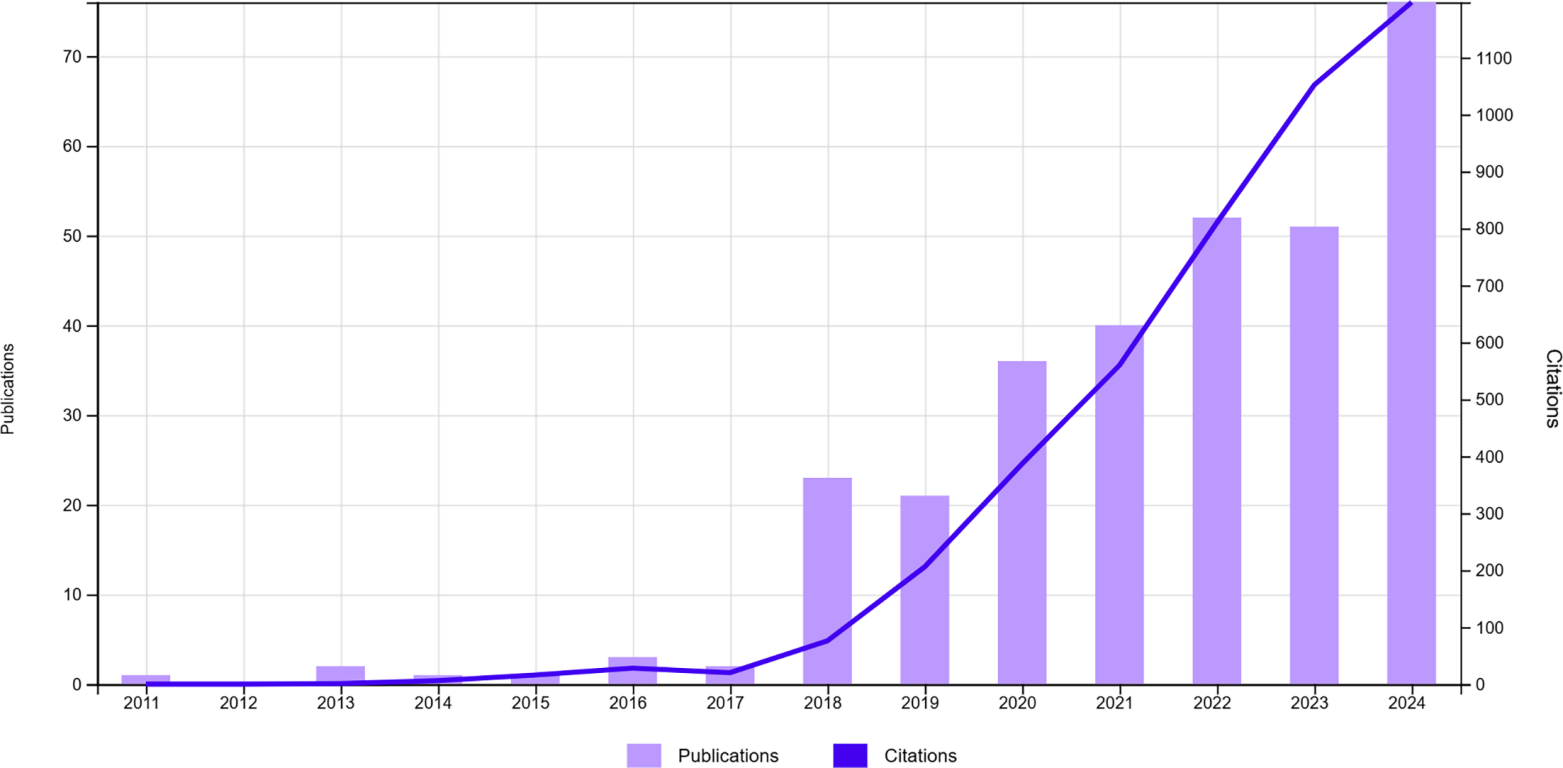
Distribution of the publications about the relationship between urinary system cancers and microbiota and the number of citations to the publications by year.

As a result of the search in WOS, a total of 310 studies investigating the relationship between bladder, prostate and kidney tumours and microbiota or microbiomes were found. Congress abstracts made up 36.77% of the publications, followed by research articles (36.13%) and reviews (18.39%). Three hundred one of the papers appeared in Science Citation Index Expanded journals, while 70 in Conference Proceeding Citation Index and 9 in Emerging Source Citation Index. The first publication on the subject entitled ‘Urolithin A and Ellagic Acid Inhibit Prostate Cancer Through Different Molecular Mechanisms: Implications of Gut Microbiome Metabolism for Cancer Prevention’ was published in 2011 by Vicinanza *et al*. [8]. Until 2018, the number of publications in a year did not exceed three, while there has been a logarithmic increase in the number of publications since 2018. The highest number of publications was reached in 2024 with 77 publications. 2024 was followed by 2022 with 52 publications and 2023 with 51 publications (Fig. 1).

The number of publications and the number of citations exhibited an equivalent pattern. Since 2018, the number of citations has increased logarithmically, similar to the number of documents. A total of 4,380 citations have been made to 310 publications thus far. The total number of citations decreased to 3,299 when self-citations were excluded. The average number of citations per publication was 14.13. The document of Sfanos *et al.* in 2018 entitled ‘The inflammatory microenvironment and microbiome in prostate cancer development’ ranked first with 296 citations [9]. It was followed by the publication of Bučević Popović *et al.* in 2018 with 170 citations and the publication of Shrestha *et al.* in 2018 with 167 citations [10,11]. Furthermore, the document by Sfanos *et al*. was placed in the first rank in terms of citations per year, with 42.29 citations. The imprint information of the ten most cited publications is given in Table 1.

**Table 1.** The detailed information about the most cited documents about the connection between urinary system cancers and microbiota

	**Title of the document**	**Journal**	**Author**	**Publication year**	**Country/department of the first author**	**Number of citation**	**Average citations per year**	**Ref**.
**1**	The inflammatory microenvironment and microbiome in prostate cancer development	*Nature Reviews Urology*	Sfanos K, Yegnasubramanian S, Nelson W, de Marzo AM	2018	USA/Pathology	296	42.29	[9]
**2**	The urinary microbiome associated with bladder cancer	*Scientific Reports*	Bučević Popović V, Šitum M, Chow CET, Chan LS, Roje B, Terzić J	2018	Croatia/Chemistry	170	24.290	[10]
**3**	Profiling the Urinary Microbiome in Men with Positive versus Negative Biopsies for Prostate Cancer	*Journal of Urology*	Shrestha E, White JR, Yu SH, Kulac I, Ertunc O, De Marzo AM, Yegnasubramanian S, Mangold LA, Partin AW, Sfanos KS	2018	USA/Pathology	167	23.86	[11]
**4**	Profiling the Urinary Microbiota in Male Patients With Bladder Cancer in China	*Frontiers in Cellular and Infection Microbiology*	Wu P, Zhang G, Zhao J, Chen J, Chen Y, Huang W, Zhong J, Zeng J	2018	China/Urology	153	21.86	[31]
**5**	The Role of Gut Microbiome in the Pathogenesis of Prostate Cancer: A Prospective, Pilot Study	*Urology*	Golombos DM, Ayangbesan A, O'Malley P, Lewicki P, Barlow L, Barbieri CE, Chan C, DuLong C, Abu-Ali G, Huttenhower C, Scherr DS	2018	USA/Urology	142	20.29	[32]
**6**	The microbiome in prostate inflammation and prostate cancer	*Prostate Cancer and Prostatic Diseases*	Porter CM, Shrestha E, Peiffer LB, Sfanos KS	2018	USA/Pathology	133	19	[33]
**7**	Stool Microbiome Profiling of Patients with Metastatic Renal Cell Carcinoma Receiving Anti-PD-1 Immune Checkpoint Inhibitors	*European Urology*	Salgia NJ, Bergerot PG, Maia MC, Dizman N, Hsu J, Gillece JD, Folkerts M, Reining L, Trent J, Highlander SK, Pal SK.	2020	USA/Medical Oncology	117	23.4	[34]
**8**	Metabolic Biosynthesis Pathways Identified from Fecal Microbiome Associated with Prostate Cancer	*European Urology*	Liss MA, White JR, Goros M, Gelfond J, Leach R, Johnson-Pais T, Lai Z, Rourke E, Basler J, Ankerst D, Shah DP	2018	USA/Urology	113	16.14	[35]
**9**	Gut Microbiota-Derived Short-Chain Fatty Acids Promote Prostate Cancer Growth via IGF1 Signaling	*Cancer Research*	Matsushita M, Fujita K, Hayashi T, Kayama H, Motooka D, Hase H, Jingushi K, Yamamichi G, Yumiba S, Tomiyama E, Koh Y, Hayashi Y, Nakano K, Wang C, Ishizuya Y, Kato T, Hatano K, Kawashima A, Ujike T, Uemura M, Imamura R, Rodriguez Pena MDC, Gordetsky JB, Netto GJ, Tsujikawa K, Nakamura S, Takeda K, Nonomura N	2021	Japan/Urology	100	25	[36]
**10**	Compositional differences in gastrointestinal microbiota in prostate cancer patients treated with androgen axis-targeted therapies	*Prostate Cancer and Prostatic Diseases*	Sfanos KS, Markowski MC, Peiffer LB, Ernst SE, White JR, Pienta KJ, Antonarakis ES, Ross AE	2018	USA/Pathology	97	13.86	[37]

When the distribution of the publications by country was analysed, the USA ranked first with 98 publications (31.61%), followed by China with 60 publications (19.35%) and Italy with 31 publications (10%). When the number of citations was analysed, the USA ranked first again with 2,523 citations, accounting for 57.6% of all citations. The USA was followed by China (875, 19.98%) and Italy (508, 11.6%). When the number of citations per document was analysed, the USA ranked first with 25.74, followed by Canada with 24.38 and Italy with 16.39, while China ranked fourth with 14.58. The number of publications, total number of citations, number of citations excluding self-citations, number of citations per document and H-indices of countries are given in Table 2.

**Table 2.** The number of publications, the total number of citations, the number of citations per document, and the H-indices of the most published 12 countries

No.	Country	Document		Citation		Without self-citation		Average citations per document	H-index
		*N*	%	*N*	%	*N*	%		
**1**	**USA**	98	31.61	2,523	57.60	2357	71.45	25.74	29
**2**	**China**	60	19.35	875	19.98	801	24.28	14.58	17
**3**	**Italy**	31	10	508	11.6	489	14.82	16.39	10
**4**	**Japan**	28	9.03	264	6.03	254	7.70	9.43	6
**5**	**Canada**	13	4.19	317	7.24	314	9.52	24.38	7
**6**	**South Korea**	11	3.55	33	0.75	31	0.94	3	4
**7**	**France**	10	3.23	92	2.1	91	2.76	9.2	5
**8**	**Poland**	10	3.23	113	2.58	111	3.36	11.3	4
**9**	**England**	9	2.90	85	1.94	85	2.58	9.44	4
**10**	**Brazil**	7	2.26	133	3.04	132	4.00	19	3
**11**	**India**	6	1.94	16	0.37	15	0.45	2.67	2
**12**	**Taiwan**	4	1.29	37	0.84	37	1.12	9.25	2
	**All**	310	100	4,380	100	3,299	100	14.13	37

The *Journal of Urology* was the journal with the highest number of publications about the relationship between urinary system cancers and microbiota, with 28 (9.03%) publications, followed by the *Journal of Clinical Oncology* with 23 (7.42%) publications and *Cancer Research* with 22 (7.1%) publications. The most cited journal was *European Urology* with 240 (5.48%) citations, followed by *Journal of Urology* with 233 (5.32%) citations and *Cancer Research* with 100 (2.28%) citations. The ten journals with the highest number of publications are given in Table 3.

**Table 3.** The number of citations, the number of citations per document and the H-indices of the ten most published journals about urinary tract cancers and microbiota

No.	Journal	Document		Citation		Without self-citation		Average citations per document	H-index
		*N*	%	*N*	%	*N*	%		
1	*Journal of Urology*	28	9.03	233	5.32	231	7	8.32	4
2	*Journal of Clinical Oncology*	23	7.42	79	1.80	79	2.39	3.43	4
3	*Cancer Research*	22	7.10	100	2.28	100	3.03	4.55	1
4	*European Urology*	15	4.84	240	5.48	238	7.21	16	4
5	*Cancers*	11	3.55	163	3.72	156	4.73	14.82	5
6	*International Journal of Molecular Sciences*	9	2.90	70	1.6	70	2.12	7.78	3
7	*Cancer Science*	8	2.58	53	1.21	52	1.58	6.63	1
8	*Urologic Oncology Seminars and Original Investigations*	7	2.26	93	2.12	91	2.76	13.29	3
9	*International Journal of Urology*	6	1.94	53	1.21	52	1.58	8.83	1
10	*Nature Reviews Urology*	6	1.94	394	9	393	11.91	65.67	3
11	*All*	310	100	4,380	100	3,299	100	14.13	37

**Fig. 2.**
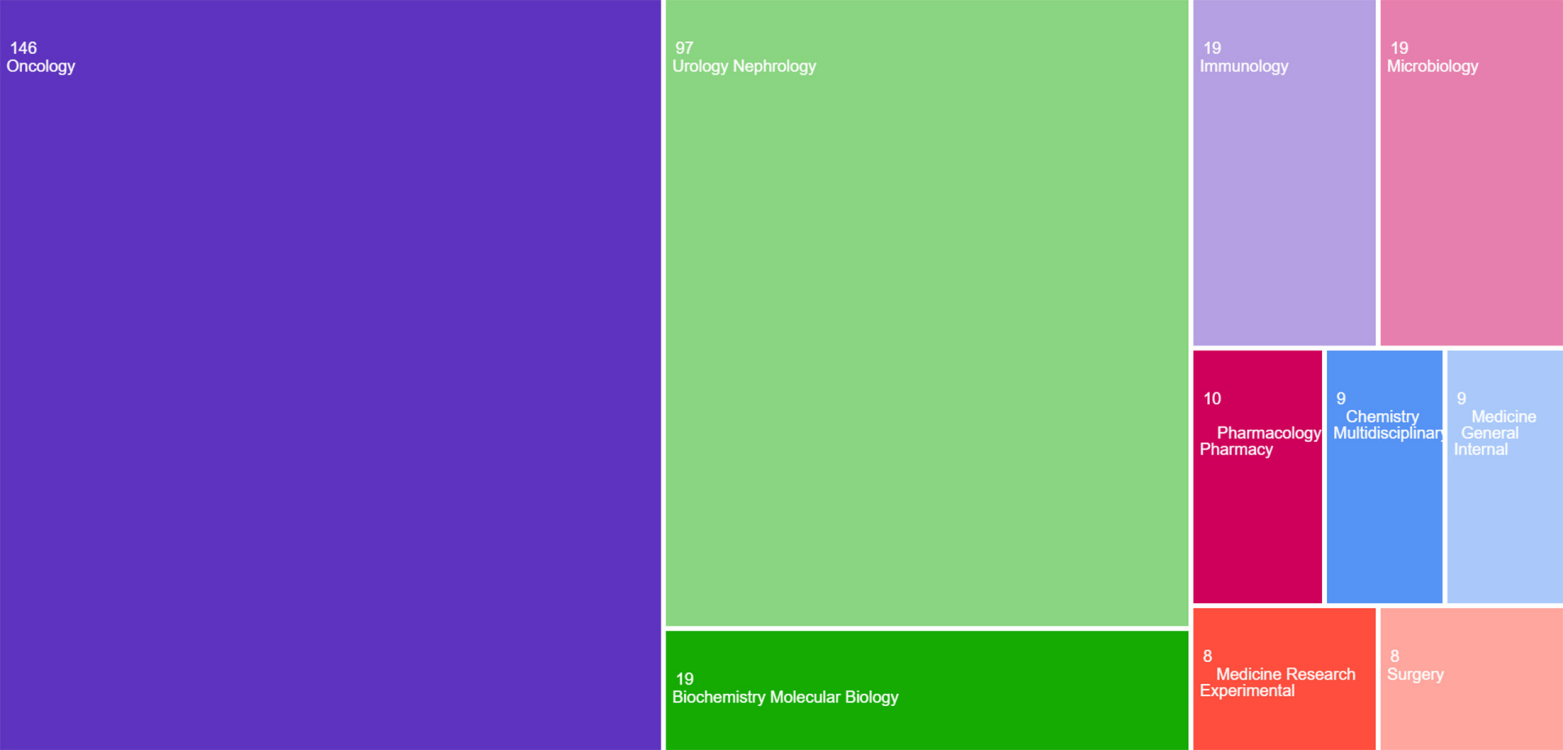
The ten most published departments about the relationship between urinary system cancers and microbiota.

When the departments with the highest number of publications were analysed, oncology ranked first with 146 publications, followed by urology–nephrology with 97 publications and biochemistry–molecular biology with 19 publications. The ten departments in which 78.71% of the publications were made are given in Fig. 2.

**Fig. 3.**
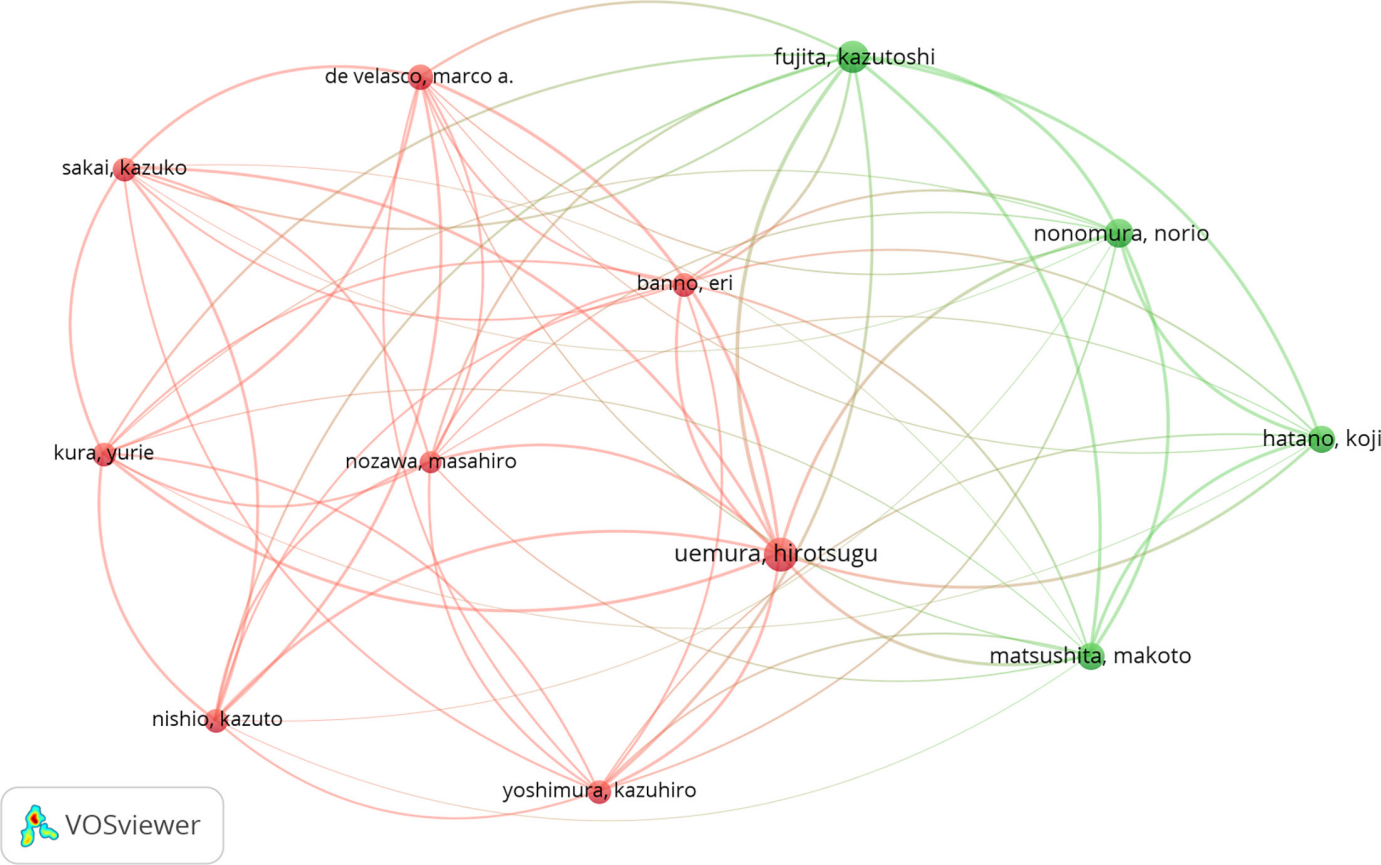
Authors with the highest number of publications on the relationship between urinary system cancers and microbiota and their collaborations (the authors were divided into two clusters and coloured differently. The size of the node indicates the number of publications of the authors).

The author with the highest number of publications on the relationship between urinary system cancers and microbiota was Hirotsugu Uemura, with 19 publications; Norio Nonomura ranked second with 17 publications, and Marco A. De Velasco ranked third with 15 publications. The authors with the highest number of publications on the subject and their collaborations are given in Fig. 3.

**Fig. 4.**
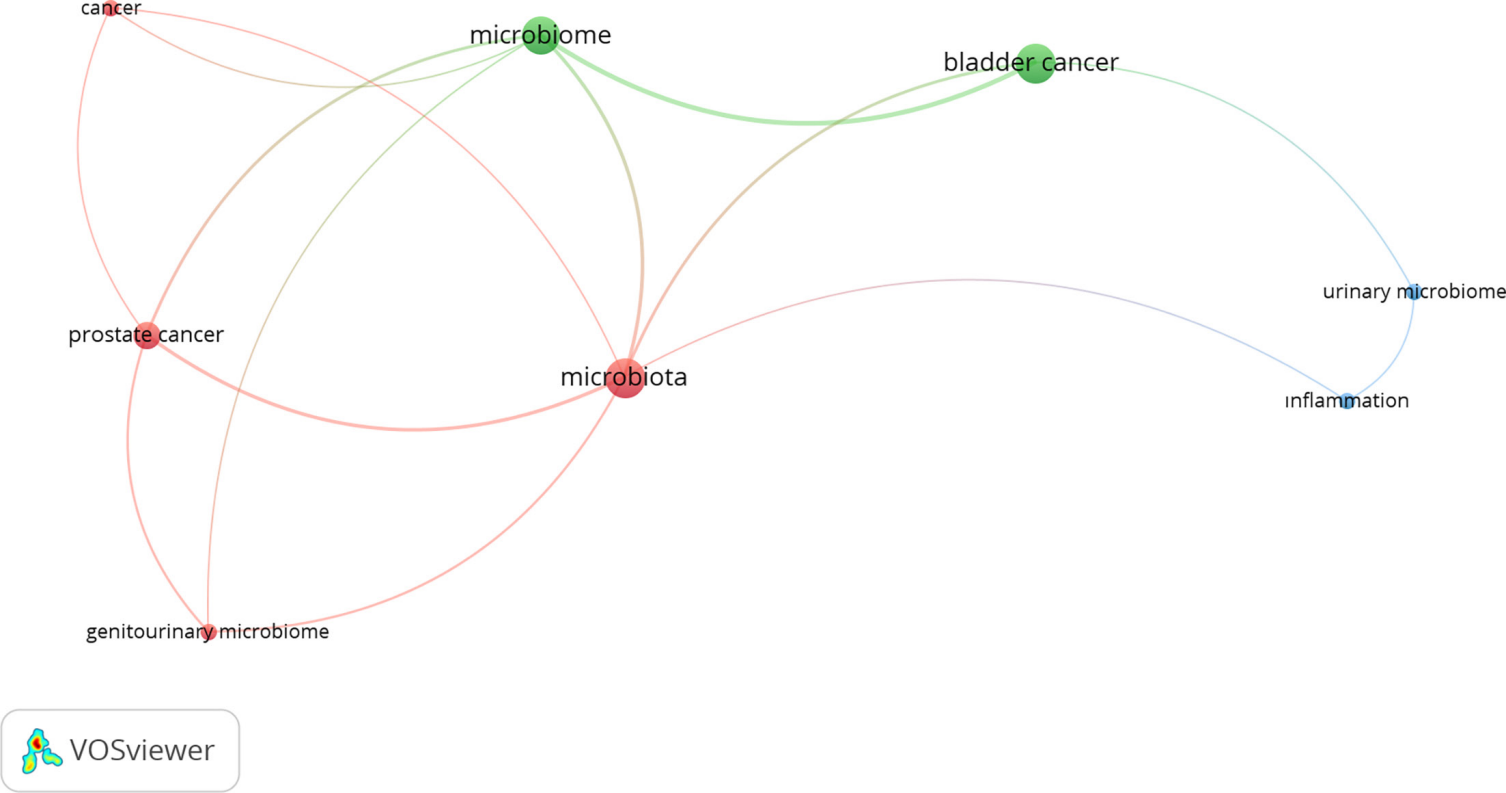
The most repetitive keywords appeared in documents about urinary system cancers and microbiota (the keywords were divided into three categories and coloured differently. The sizes of the nodes indicate how many publications used that term).

The most frequently appearing keywords in publications were microbiota and bladder cancer, with 11 repetitions, followed by microbiome with 10 repetitions and prostate cancer with 5 repetitions (Fig. 4).

## Discussion

The number of studies investigating the relationship between microbiota and cancer is increasing day by day. The relationship between microbiota and cancer is established through pathogenesis, prognosis and treatment. Micro-organisms have been reported to be responsible for ~20% of human malignancies [12]. Metabolites of micro-organisms can cause changes in the proliferation–death balance of host cells in inflammatory processes [13]. In addition, in case of dysbiosis, which means disruption of the microbiota, an increase in pathogenic micro-organisms that cause carcinogenesis can be replaced by the symbiotic micro-organisms [14,15]. It has been reported that changes in the gut microbiota, which has the richest micro-organism content in the human body with ~1,000 bacterial species, can also affect extraintestinal regions through systemic effects [16,17]. Prostate cancer, bladder cancer and kidney cancer, the most common urologic malignancies, account for 9.8% of all cancer incidence worldwide and 9.1% of tumour-related deaths [18]. A recent study supported a causal relationship between gut microbiota and prostate cancer, bladder cancer and kidney cancer, with different bacterial characteristics being identified in relation to each tumour type [19].

Although the gut microbiota was discovered in 1944, it took nearly 60 years before studies on the microbiota–cancer relationship were published [20]. The first study indexed in WOS about the relationship between microbiota and cancer was conducted in 2002, and the relationship between microbiota and oral cancers was investigated [21]. On the other hand, 9 years later, in 2011, the first study examining the connection between urologic malignancies and microbiota was published. Vicinanza *et al*. [8] studied the potential of gut microbiota metabolites called urolithin A and ellagic acid to prevent prostate cancer. While studies investigating the relationship between urologic cancer and microbiota increased at a low rate until 2018, the rapid acceleration of these studies since 2018 is undoubtedly related to the concentration of interest in this field and the acceleration of the increase in knowledge. At this point, it is necessary to draw attention to the Human Microbiome Project (HMP), which was initiated in 2007 and completed in 2016. The first phase of the HMP, analysing the genetic profiles of micro-organisms from various parts of the human body, was completed in 2014. With the development of metatranscriptomic, metabolomic and proteomic methods, the Integrative HMP phase, in which conditions such as type 2 diabetes, inflammatory bowel disease and preterm labour were tried to be associated with microbiota, was completed in 2016 [22]. The highest number of publications on the relationship between urologic cancer and microbiota was reached in 2024, and studies are likely to increase even faster after that.

A total of 310 publications indexed in WOS investigating the relationship between urologic cancers and microbiota were identified. Studies investigating the relationship between microbiota and bladder, kidney and prostate cancers, which are among the most common urologic cancers, were conducted most frequently in the USA, with a rate of 31.61%, followed by China, Italy and Japan (Table 2). The top three countries that contribute most to the studies evaluating microbiota and cancer relationships in the last 20 years have been the USA, China and Italy, respectively, compatible with the most contributing countries to the publications about urological cancers and microbiota relationships in our study [23]. According to GLOBOCAN data, bladder cancer accounts for ~3% of all cancer cases, and bladder cancers are mostly seen in developed countries. The fact that bladder cancer cases are frequently found in the south and west of Europe and the north of the USA may explain why most of the studies were conducted in the USA and Italy [24]. In another study, it was announced that the incidence of bladder cases in 2019 was highest in Japan, and disability-adjusted life year (DALY) rates were highest in China [25]. The countries with the highest incidence of renal tumours are the USA, China and Japan [26]. The USA, Japan, China and Italy are also countries where prostate cancer is common [27]. The fact that most of the studies about the relationship between urologic cancers and microbiota were conducted in the USA, China, Italy and Japan may be associated with the higher incidence of cancer types in these countries or the high rate of DALY loss in these countries.

The USA was the top-ranked country in studies examining the connection between urologic malignancies and microbiota, but the first two authors who published the most documents were from Japan. Hirotsugu Uemura was the leader with 19 publications. All of the publications were about the connection between gut microbiota and prostate cancer. Norio Nonomura, who ranked second in terms of the number of publications, had 17 publications. Sixteen of his publications were related to prostate cancer and gut microbiota, while one study was related to bladder cancer and microbiota. Of the publications indexed in WOS, two of the most cited publications were found to be related to bladder cancer and two to prostate cancer. Consistent with this, in studies investigating the relationship between urologic cancer and microbiota, prostate cancer was the leader with a rate of 45.48%, followed by bladder cancer with 36.77%. Studies evaluating the relationship between renal tumours and microbiota constitute 17.64% of all studies, and there was only one renal tumour-related document among the top ten most cited studies (Table 1). The scarcity of studies on renal tumour microbiota and the fact that they are cited less suggest that the renal tumour microbiota relationship remains less prioritized compared to others. This suggests that among bladder, prostate and renal tumours, renal tumours are the least emphasized in relation to microbiota. The fact that the studies investigating the relationship between bladder and prostate cancers and microbiota were more than the studies investigating the relationship between renal tumours and microbiota was also reflected in the most frequently mentioned keywords in the publications. In the most frequently mentioned keywords in publications, ‘microbiota’ and ‘bladder cancer’ ranked first with 11 repetitions, ‘microbiome’ ranked second with 10 repetitions and ‘prostate cancer’ ranked third with 5 repetitions (Fig. 4).

Since the 2000s, innovations in artificial intelligence, microbiome analysis and new-generation sequencing techniques have made it feasible to generate more data more quickly. As first-, second- and third-generation sequencing technologies have advanced, the technology’s cost has decreased [28]. Along with the advancement of sequencing techniques, the completion of the HMP has accelerated research about the microbiota of cancer patients. The discovery of microbiota variations is thought to have applications in early diagnosis, treatment or prognosis monitoring [29]. Recent research on the connection between microbiota and urological cancers, particularly prostate and bladder, indicates that the knowledge gathered from these investigations will be utilized to decrease the morbidity and mortality of cancer patients in a short time. Microbiota research has generally been advanced by national efforts conducted independently so far. However, they ought to be transformed into global strategies that will use samples that are highly representative of the universe and compare them to the control group with repeated samples during the follow-up period to comprehend the role of the microbiota in the development of cancer and use them as therapeutic approaches [30].

WOS is a comprehensive, valuable database containing high-quality and reputable journals, and it is thought that the analysis of the studies investigating the relationship between urological cancers and microbiota indexed in the WOS database for the first time will contribute to the literature. However, the use of WOS as the only database in our study can also be considered a limitation of the study.

## Conclusion

Although the discovery of the microbiota dates back 80 years, with the completion of the second phase of the HMP, iHMP, in 2016, there has been a rapid increase in the number of studies trying to detect microbiota differences in various disease states and investigating some microbiota-regulating applications in the treatment of diseases. Similarly, the number of studies investigating the relationship between urological cancers and microbiota has started to increase with acceleration since 2018 and peaked in 2024. It seems that microbiota will gain more importance in the ongoing process, and the number of these studies will continue to increase. In the presented study, a bibliometric analysis of the studies investigating the relationship between prostate, bladder and renal tumours and microbiota was performed, and to the best of our knowledge, this is the first bibliometric study on this subject in the literature so far. Among these three cancer groups, the number of studies investigating the relationship between prostate and bladder cancers and microbiota was found to be higher than renal tumours; therefore, the relationship between bladder and prostate cancers and microbiota came to the fore in the bibliometric analysis. Although it is clear that more research is required on this topic, it is hoped that the identification of microbiota variations in cancer types may eventually be used in early diagnosis, prognosis assessment and treatment.

## Abbreviations

DALY: disability-adjusted life year
HMP: Human Microbiome Project
RCC: renal cell carcinoma
WOS: Web of Science
